# CRISPR-dCas9 and sgRNA scaffolds enable dual-colour live imaging of satellite sequences and repeat-enriched individual loci

**DOI:** 10.1038/ncomms11707

**Published:** 2016-05-25

**Authors:** Yi Fu, Pedro P. Rocha, Vincent M. Luo, Ramya Raviram, Yan Deng, Esteban O. Mazzoni, Jane A. Skok

**Affiliations:** 1 Department of Pathology, New York University School of Medicine, 550 First Avenue, Sml 311, New York, New York 10016, USA; 2 Department of Biology, New York University, New York, New York 10003, USA; 3 Microscopy Core, New York University School of Medicine, New York, New York 10016, USA

## Abstract

Imaging systems that allow visualization of specific loci and nuclear structures are highly relevant for investigating how organizational changes within the nucleus play a role in regulating gene expression and other cellular processes. Here we present a live imaging system for targeted detection of genomic regions. Our approach involves generating chimaeric transcripts of viral RNAs (MS2 and PP7) and single-guide RNAs (sgRNAs), which when co-expressed with a cleavage-deficient Cas9 can recruit fluorescently tagged viral RNA-binding proteins (MCP and PCP) to specific genomic sites. This allows for rapid, stable, low-background visualization of target loci. We demonstrate the efficiency and flexibility of our method by simultaneously labelling major and minor satellite regions as well as two individual loci on mouse chromosome 12. This system provides a tool for dual-colour labelling, which is important for tracking the dynamics of chromatin interactions and for validating epigenetic processes identified in fixed cells.

## Abstract (web summary)

The ability to target Cas9 to specific genomic loci offers the potential for applications beyond genome editing. Here the authors use deactivated Cas9 and chimeric guide RNAs to visualise individual repetitive loci in living cells.

## Graphical abstract

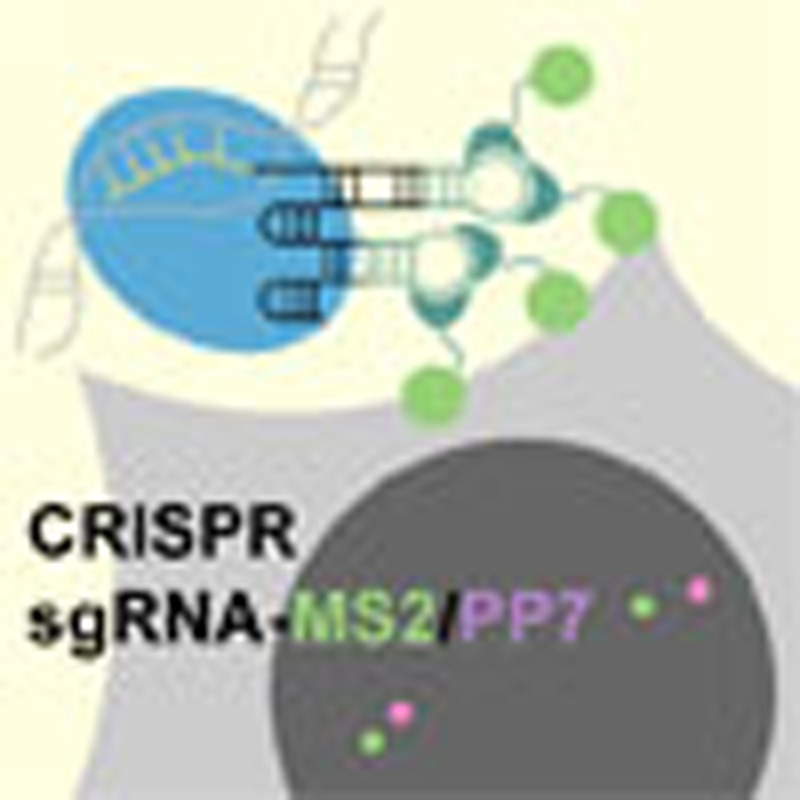

Chromatin dynamics and nuclear localization contribute to spatiotemporal gene regulation, which is important for the coordination of developmental processes, such as lineage and stage-specific gene activation and repression, DNA replication, DNA damage repair and programmed DNA rearrangement during lymphocyte development^1^,^2^,^3^,^4^,^5^,^6^,^7^. Most investigations tracking the dynamics of specific loci have made use of FISH (fluorescent *in situ* hybridization) in fixed cells. Although we have learned much from this approach, the analysis only provides a snapshot of events that occur in single cells, leaving gaps in our understanding of the processes being studied^8^. The only way to obtain a complete picture of chromatin dynamics is to use a live imaging system. Until recently, live imaging approaches have largely been restricted to the use of fluorescently tagged DNA-binding proteins (such as LacR or TetR), which although extremely robust, require laborious gene targeting and insertion of large LacO and TetO DNA arrays that could potentially affect the chromatin structure and function of targeted loci^9^,^10^,^11^.

The recent discovery of engineered endonucleases for gene targeting has revolutionized the field of live imaging. Specifically, adaptation of the type II (clustered regularly interspaced short palindromic repeats) CRISPR system using a nuclease defective Cas9 (dCas9) fused to a fluorescently labelled protein have made it possible to label specific genomic loci by designing single-guide RNAs (sgRNAs) that will recruit the tagged dCas9 protein to the DNA^12^,^13^,^14^. Recruitment of dCas9 depends on the presence of a short 20-nucleotide sequence at the 5′-end of the sgRNA, which is complementary to the sequence of the target site. This makes the cloning of locus-specific sgRNAs trivial and the system easy to implement. Target loci need to be immediately followed by a protospacer adjacent motif (PAM) that can differ between bacterial species. For *Streptococcus pyogenes* Cas9, the PAM consists of the NGG sequence, a motif that is found at high frequency in the genome enabling recruitment to virtually any genomic location.

An essential feature of any live imaging system is the ability to visualize more than one locus at a time. A common approach to deal with this problem is to tag dCas9 proteins from different bacterial species with different fluorescent proteins^15^,^16^. As each bacterial Cas9 recognizes specific PAMs sequences, different loci can be simultaneously labelled. One problem with this system, however, is that other bacterial Cas9 proteins (such as from *Neisseria meningitides, Streptococus thermophiles* or *Staphylococcus aureus*) recognize longer PAM sequences and these are therefore less flexible for target design. In addition, the large size of fluorescently tagged dCas9 proteins increases the difficulty of transfection or viral infection as multiple large fusion proteins have to be introduced in the same eukaryotic cells. An alternative approach to achieve dual-colour labelling has been to use transcription activator-like effectors fused to fluorescent proteins^17^. Nonetheless, compared with the CRISPR system, TAL technology is less flexible and more laborious. This is particularly pertinent for labelling of unique, non-repetitive genomic regions where numerous sgRNA targets (a minimum of 36 (ref. 12)) have to be designed for a short region to ensure that sufficient fluorescently labelled proteins are recruited.

To circumvent these issues and to enable simultaneous visualization of two distinct genomic regions, we generated sgRNAs containing the RNA stem-loop motifs MS2 and PP7 that are bound by the bacteriophage coat proteins MCP (MS2 coat protein) and PCP (PP7 coat protein), respectively^18^,^19^. These sgRNAs are recruited to two distinct target regions by dCas9 and visualized with fluorescent proteins tagged to the RNA-binding viral proteins, mCherry for PCP and enhanced green fluorescent protein (EGFP) for MCP. As a proof-of-principle, we simultaneously label major and minor satellite regions of murine genomes as well as two individual loci on mouse chromosome 12.

## Results

### Components of the dual-colour live imaging system

**Figure 1:**
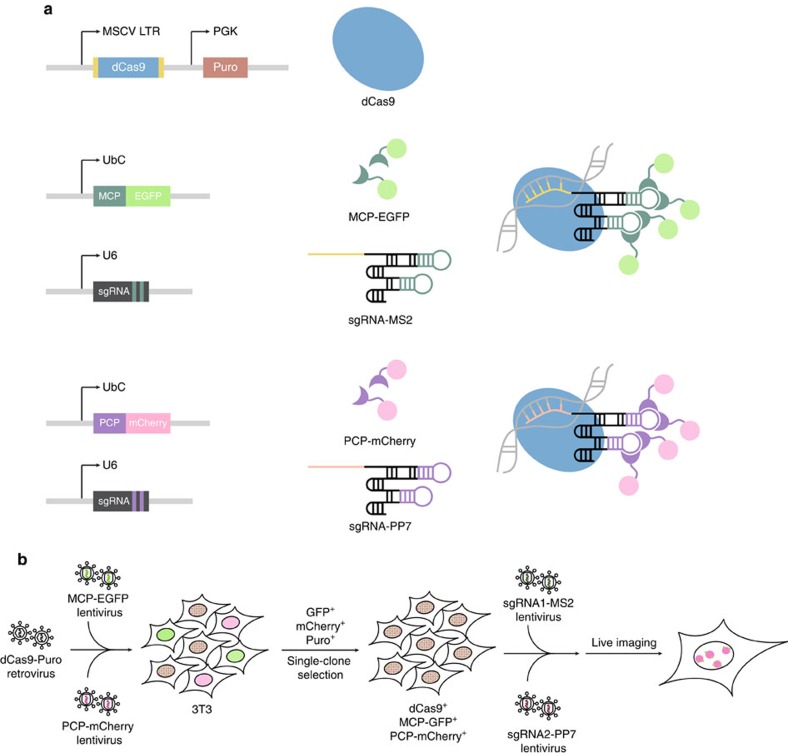
Components of the dual-colour live imaging system. (**a**) A dCas9 protein surrounded by two nuclear localization signals (in yellow) was expressed from a retroviral plasmid containing a puromycin resistance gene. sgRNAs were expressed from a U6 promoter and these could either be transfected or delivered via lentivirus. The sgRNA scaffolds contain double PP7 or MS2 stem loops. The proteins MCP and PCP fused to EGFP or mCherry, respectively, were expressed from lentiviral plasmids. dCas9 allows binding of sgRNAs to their target DNAs and sgRNAs containing MS2 or PP7 stem loops can each recruit four molecules of MCP-EGFP or PCP-mCherry, respectively. (**b**) Workflow for dual-colour live imaging. To test the system, 3T3 fibroblasts were co-infected by the dCas9 retrovirus and with the MCP-EGFP and PCP-mCherry lentiviruses, followed by puromycin selection and FACS for EGFP and mCherry double positive cells. Single clones based on low background expression of fluorescent proteins and high efficiency of labelling were selected. Optimal clonal cells can then be transduced or transfected with plasmids carrying sgRNAs for subsequent live imaging.

The system we present here is composed of three different components: (i) a deactivated Cas9 (dCas9), (ii) sgRNAs targeting two loci of interest that contain either the MS2 or the PP7 stem loops embedded in their scaffolds and (iii) RNA-binding proteins (MCP and PCP) fused to fluorescent proteins (Fig. 1a). The dCas9 protein, which is necessary for hybridization of sgRNAs to target DNA, is surrounded by two nuclear localization signals that ensure proper nuclear import. It is expressed from a retroviral construct containing a puromycin resistance gene^14^.

Expression of the sgRNAs is driven by a mouse U6 promoter incorporated within lentiviral plasmids. The sgRNA scaffolds contain two copies of the MS2 or PP7 stem-loop motifs extended from the tetraloop and stem loop (Fig. 1a). These two regions of the sgRNA scaffold have been shown to protrude out of Cas9–sgRNA complex. Thus, addition of MS2 or PP7 to these regions has little impact on Cas9 function. We used an sgRNA-MS2 chimaeric transcript design that the Zhang lab has shown to be highly efficient for recruiting transcription co-factors via MCP^20^. As for the sgRNA-PP7 RNA we designed our own construct to generate a secondary RNA structure closely resembling its MS2 counterpart. sgRNAs targeting different loci can be generated with a simple one-step cloning procedure that is amenable to high-throughput scaling.

The MS2 RNA stem loop is recognized by MCP, whereas PP7 is recognized by the PCP. These two proteins are fused to either EGFP (for MCP) or mCherry (for PCP) and expressed from lentiviral plasmids^21^. As MCP and PCP dimerize to bind to corresponding RNA motifs, each sgRNA scaffold can recruit four molecules of fluorescent protein.

The three components of the system can either be introduced simultaneously or in a stepwise manner in cell lines (Fig. 1b). In the latter case, the dCas9, MCP-EGFP and PCP-mCherry viral constructs are first used to transduce the cell line of choice to generate a stable cell line. These cells can then be treated with puromycin to select for dCas9. Subsequent fluorescence-activated cell sorting (FACS) can be used on dCas9-positive cells to isolate those cells that express both fluorescently labelled proteins at low intensity. The establishment of clonal cell lines enables identification of cells that express optimal levels of fluorescent proteins. This is important as cells expressing extremely high levels of fluorescent proteins have a much higher background, which is not optimal for visualization of target loci. Selected clones can then be transfected with sgRNA constructs or alternatively infected with sgRNA lentivirus for permanent labelling. Loci targeted by sgRNAs containing the MS2 motif will be labelled in GFP, whereas the PP7 motif will allow visualization in mCherry.

### Labelling of major satellite regions using sgRNA scaffolds

**Figure 2:**
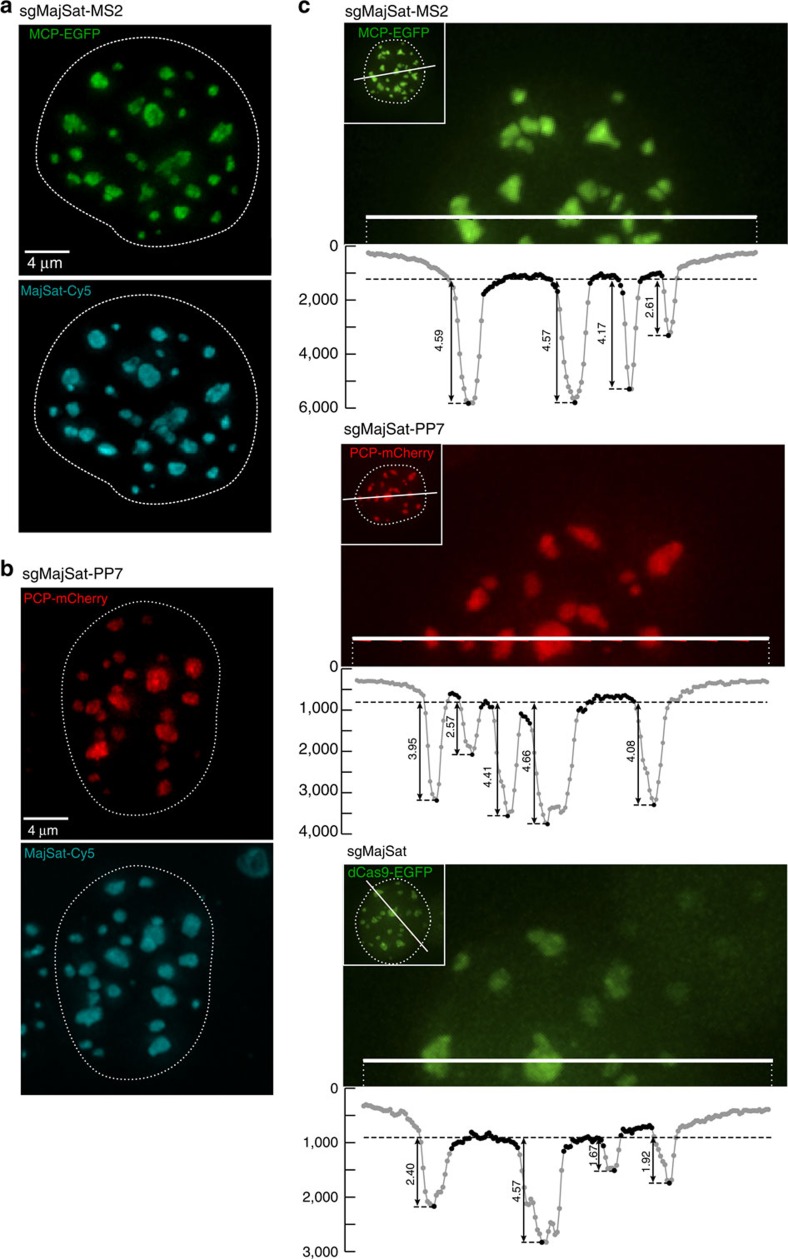
Chimaeric sgRNAs allow recruitment of fluorescent proteins and efficient labelling of major satellites. (**a**,**b**) Expression of either sgMajSat-MS2 or sgMajSat-PP7 sgRNAs in cells expressing dCas9, MCP-EGFP and PCP-mCherry leads to efficient labelling of pericentromeric regions in green or red, respectively (top panels). Fluorescent protein signals were validated using FISH with a γ-satellite probe (bottom panels). (**c**) The signal obtained with sgRNAs was compared with dCas9-EGFP fusion protein signal. Signal to background ratio was measured from raw images. The long dashed line across the entire plot represents the averaged intensity of background. The short dashed lines indicate the highest value of each peak, which is the highest intensity of each PCH punctum. Values next to vertical arrows represent the signal-to-background ratio.

To test our method, we used the 3T3 mouse fibroblast cell line and infected these with dCas9-, MCP-EGFP- and PCP-mCherry-expressing viruses. Upon puromycin selection, we derived clones with low background expression of the fluorescent proteins (Supplementary Fig. 1a). We then designed an sgRNA targeting the murine major satellite region (sgMajSat-MS2), which consists of a 234-bp repeat unit spanning 6 Mb of pericentromeric DNA. Major satellites form a three-dimensional (3D) nuclear structure known as pericentromeric heterochromatin (PCH), which is easily identified as large 4,6-diamidino-2-phenylindole (DAPI) bright heterochromatic domains. We first expressed the sgMajSat-MS2 sgRNA with the RNA scaffold designed by the Zhang lab containing two copies of the MS2 stem-loop domain^20^. The sgMajSat-MS2 was able to efficiently recruit MCP-EGFP protein to the PCH region in the 3T3 cell line (Fig. 2a, top panel). To validate the MCP-EGFP signal, we performed FISH using a plasmid containing eight copies of the 234-bp major satellite repeat as a probe. FISH and MCP-EGFP signals were perfectly matched as shown in the bottom panel of Fig. 2a. Cells not transfected with the sgMajSat-MS2 construct do not highlight major satellite-like structures (Supplementary Fig. 1a).

We next designed an sgRNA scaffold containing 2 PP7 stem loops (that mimic the structure of sgRNA-MS2) for recruitment of the PCP-mCherry protein. Two different sequences of the *PP7* motif predicted to form the same secondary structure were tested (Supplementary Fig. 2)^22^. To visualize recruitment of PCP-mCherry by these constructs, we again used an sgRNA targeting the major satellite sequence (sgMajSat-PP7). In contrast to the MCP-EGFP protein, PCP-mCherry localizes at the cell nucleolus in the absence of sgRNAs (Supplementary Fig. 1a). However, upon sgMajSat-PP7 expression, PCP-mCherry was readily recruited to PCH regions (Fig. 2b). We also validated our signal using FISH, which demonstrated the appropriate recruitment of PCP-mCherry at pericentromeric regions. Both versions of the PP7 construct achieved similar levels of PCH labelling efficiency, according to the residual accumulation of PCP-mCherry at nucleoli (Supplementary Fig. 2). Importantly, we verified that there is no cross recruitment of MCP-EGFP protein to PP7 stem loops and PCP-mCherry protein to MS2 motifs (Supplementary Fig. 1b).

To compare our system with previous CRISPR-dCas9-EGFP labelling methods, we used a retroviral construct expressing EGFP-fused dCas9 with flanking nuclear localization signal (NLSs). Using FACS, we isolated cells capable of low background expression of EGFP. We then introduced a major satellite-targeting sgRNA, with a scaffold structure optimized for CRISPR imaging^12^. This sgRNA has the same major satellite target sequence and the same plasmid backbone as the sgMajSat-MS2 and sgMajSat-PP7 constructs. To assess the labelling efficiency of these three systems, we compared the intensity of fluorescent signal at PCH regions with background signal (Fig. 2c). Our chimaeric sgRNA scaffold system displayed signal to noise levels similar to that of the dCas9-EGFP fusion system demonstrating that this is a valid strategy for labelling of genomic loci.

### Dual-colour live imaging of minor and major satellites

**Figure 3:**
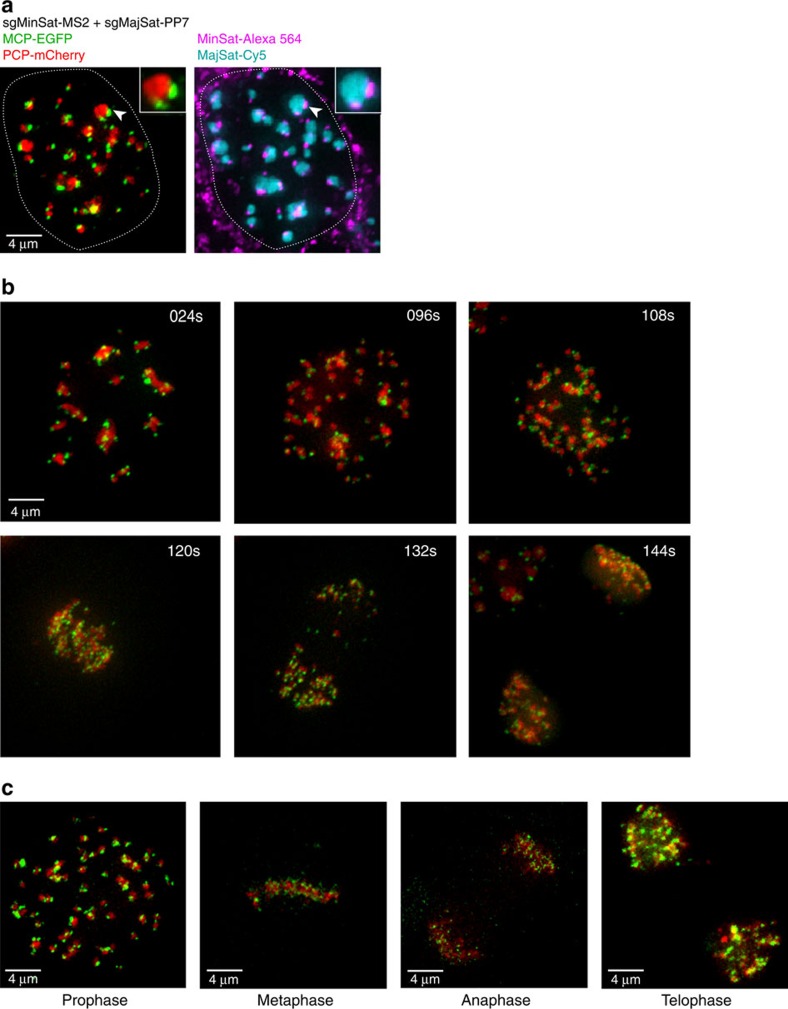
Imaging of minor and major satellite regions throughout cell cycle. (**a**) 3T3 fibroblasts expressing dCas9, PCP-mCherry and MCP-EGFP were transfected with sgMajSat-PP7 and sgMinSat-MS2. Insets shows a zoomed view of a centromeric region containing the expected signal of minor and major satellites (left panel). Fluorescent protein signals were validated by DNA FISH (right panel). (**b**) One cell with labelled minor and major satellites was followed throughout cell cycle. (**c**) Cells at different stages of mitosis were captured in time-lapse live imaging.

For simultaneous labelling of two genomic structures to complement the pericentromeric labelling, we selected the minor satellite repeats of murine chromosomes as our target sequence. Minor satellites consist of 120 bp units repeated over a 0.6–1.2 Mb region. We designed an sgRNA targeting this unit containing two MS2 motifs (sgMinSat-MS2). We then introduced the sgMinSat-MS2 sgRNA together with sgMajSat-PP7 into our 3T3 cell line expressing dCas9, MCP-EGFP and PCP-mCherry. Formation of an easily recognizable structure consisting of two smaller minor satellite signals surrounding larger major satellites was readily visible and validated by FISH analysis (Fig. 3a and Supplementary Movie 1).

Live imaging over long periods of time can only be performed with short exposure times because of the photo-toxicity resulting from excitation of fluorescent proteins. To test this and to ensure that our chimaeric sgRNAs allow tracking of genomic loci throughout cell cycle, we performed live imaging for a period of 10 h tracking the 3T3 fibroblasts transduced with both sgMinSat-MS2 and sgMajSat-PP7 (Fig. 3b and Supplementary Movie 2). With this approach, we were able to track our cells throughout cell cycle. Major and minor satellite regions displayed robust chromosomal dynamics during mitosis, which is consistent with previous FISH analyses^23^ (Fig. 3c). Importantly, following cell division, daughter cells maintain correct minor and major satellite labelling (Supplementary Movie 2).

To further test the flexibility of our system, we tried implementing it in mouse embryonic stem (ES) cells. For this, we first introduced the PCP-mCherry construct and sorted for a population of cells with lower basal expression. Then, using a single round of nucleofection, we introduced the sgMinSat-MS2 and sgMajSat-PP7 sgRNAs together with an MCP-EGFP plasmid and a dCas9 construct driven by the pCAGGS promoter. The major and minor satellite signals that we observed in 3T3 cells were readily visible in ES cells, demonstrating that our system can be easily implemented with concomitant transient delivery of its components to different cell lines (Supplementary Fig. 3).

### Live imaging of two single loci on the mouse chromosome 12

**Figure 4:**
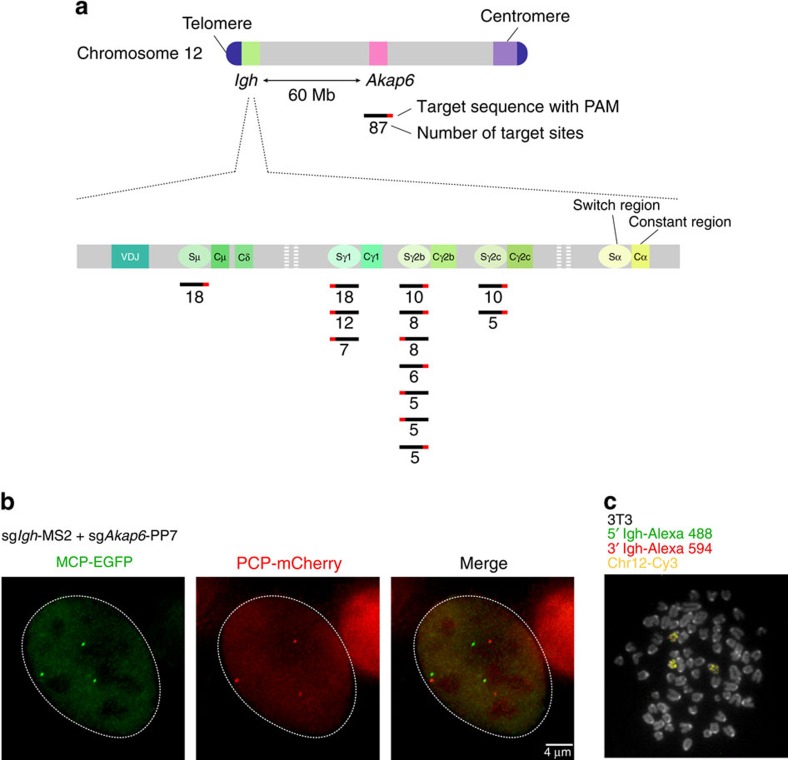
Imaging of single loci on the mouse chromosome 12. (**a**) Scheme of sgRNAs designed to target *Igh* and *Akap6* on mouse chromosome 12. A single sgRNA targets 87 locations on the *Akap6* gene across 6.2 kb. Thirteen different sgRNAs were designed to target four different *Igh* switch regions at the telomeric end of mouse chromosome 12. The number of hits for each sgRNA is written below the bar representing its location (**b**) 3T3 fibroblasts expressing dCas9, MCP-EGFP and PCP-mCherry were co-infected with virus targeting the *Akap6* gene (sg*Akap6*-PP7) and the 4 *Igh* switch regions (sg*Igh*-MS2). (**c**) Cytogenetic analysis of chromosome 12 and *Igh* on 3T3 fibroblasts using metaphase-FISH with a mouse chromosome 12 paint and 2 BAC probes surrounding *Igh (Igh* 5′ and *Igh* 3′).

To further test the flexibility and robustness of our system, we next labelled two individual loci on chromosome 12. For this, we designed sgRNAs targeting the constant region of the *imunnoglobulin heavy chain* (*Igh*) gene. The *Igh* constant region contains eight different constant region exons that allow B cells to produce antibodies with different effector functions. Preceding each of these exons are switch regions that are composed of different repeats^24^. These stretches of DNA range from 2 to 10 kb. Taking advantage of this structure, we designed 13 different sgRNA-MS2 constructs that target 4 different switch regions (Sμ, Sγ1, Sγ2b, Sγ2a) for a total of 117 predicted sgRNA-binding sites (Fig. 4a). To allow for easy validation of our *Igh* labelling, we designed one sgRNA fused with the PP7 motif targeting the *Akap6* gene located 60 Mb away from *Igh* on chromosome 12. This gene contains 209 repeats of a 29-nucleotide sequence and the sgRNA we designed is predicted to hybridize to 87 locations across a 6.3-kb region.

The sg*Igh*-MS2 and sg*Akap6*-PP7 sgRNAs were then delivered via lentiviral infection to 3T3 fibroblasts containing stable integrations of the dCas9, MCP-EGFP and PCP-mCherry contructs. Adjacent signals in different colours were readily observed following infection (Fig. 4b). As both loci are located on the same chromosome 60 Mb apart, the two signals should be closely associated without actually co-localizing. Most of the cells contained three pairs of EGFP-mCherry signals, which were validated by metaphases spread analyses. The latter show that our 3T3 cell line contains three copies of chromosome 12 and *Igh*, thereby validating the signals we observed with our live imaging system (Fig. 4c).

## Discussion

Live imaging systems for visualization of genomic loci are essential tools that are required for understanding cellular processes such as DNA replication and transcription. Here we describe a system for live imaging of specific loci that uses the flexibility of dCas9 targeting to any region of the genome that can be visualized by fluorescent RNA-binding proteins. We demonstrate that our method results in efficient and consistent labelling of nuclear structures and of individual loci that can be validated by FISH. Furthermore, we showed that we could track the dynamics of replicating centromeric regions through cell division using sgRNAs targeting the minor and major satellite regions.

Our method provides an advantage over existing dCas9-GFP systems^12^,^15^ as it allows labelling of two loci in different colours. Dual-colour labelling is essential for studies of epigenetic regulatory mechanisms that involve tracking chromatin interactions of two loci or recruitment of loci to nuclear compartments of distinct epigenetic configurations such as PCH. Another important advantage of our approach is that in contrast to existing methods that fuse fluorescent protein reporters to dCas9 of different families^15^, we use dCas9 from a single bacterial species, *S. pyogenes* (Sp). As dCas9 from other bacterial species do not contain a PAM that is as simple as the one derived from *S. pyogenes*, the use of dCas9 from different species is not so flexible as the system we describe here. Our approach can be further improved by adding a third colour. This can be achieved by designing sgRNA scaffolds containing the *com*^25^ motif, which can be recognized by a COM protein fused to BFP, thereby enabling labelling of a third locus in a different colour.

## Methods

### DNA constructs

The dCas9 retroviral plasmid (MSCVLTR-dCas9-EGFP-Puro) was obtained from Addgene (#51023)^12^. We removed the *EGFP* gene from this plasmid using *Mlu*I and *Eco*RI and added back the C-terminal of dCas9 by PCR. MCP-EGFP and PCP-mCherry plasmids were obtained from Addgene (#61764 and #61763)^21^. For nucleofection of ES cells, dCas9 and MCP-EGFP were placed in plasmids containing either a pCAGGS promoter or an EF1α promoter, respectively.

The non-viral sgRNA-MS2 backbone plasmid was obtained from Addgene (# 61424)^20^. To build the sgRNA-PP7-1 backbone plasmid, we ordered 156-nt ultramer oligos from IDT. These contain a target insertion site and the sgRNA scaffold with two PP7 motifs. The annealed DNA fragment was ligated to the sgRNA-MS2 backbone that was digested by *Bbs*I and *Eco*RI. All target sequences were cloned into sgRNA-MS2 or sgRNA-PP7-1 backbone plasmids by following the SAM protocol (http://sam.genome-engineering.org/protocols/).

Lentiviral murine-U6-driven sgRNA-MS2 and sgRNA-PP7-1 plasmids containing Major Satellite target sequences were generated by removing sgRNA-(F+E)-CMV-Puro-T2A-mCherry from plasmid pSLQ1661-sgMUC4-E3(F+E) (Addgene #51025), and replacing it with a PCR-amplified fragment of sgMajSat-MS2 and sgMajSat-PP7 from non-viral constructs. To construct lentiviral sgRNA-MS2 with different target sequences (such as MinSat and *Igh*), we digested lenti-mU6-sgMajSat-MS2 with *Bst*XI and *Eco*RI, and generated sgRNA-MS2 insertions that contain target sequences using a forward primer (5′-TTGGAGAACCACCTTGTTGG**N**_**17–21**_GTTTTAGAGCTA-3′, where N_17–21_ is the target sequence with the size of 17–21 bp). The *Bst*XI site in the forward primer is underlined. A common reverse primer (5′- GGTCCCTCGACGAATTCAAA-3′, where the *Eco*RI cut site is underlined) was used in the PCR reaction. Each forward primer was mixed with an equal amount of reverse primer to PCR amplify new sgRNA fragments using lenti-mU6-sgMajSat-MS2 as the template. The PCR reaction was performed using the Phusion HF DNA polymerase (Thermo Scientific) under the following conditions: 98 °C for 30 s; 98 °C for 20 s, 62 °C for 20 s, 72 °C for 10 s and repeated 35 times; 72 °C for 5 min; 4 °C hold. After PCR, the products were run on a 1.5% agarose gel and purified by cutting the ∼200 bp DNA band. The purified DNA was digested using *Bst*XI and *Eco*RI. The digestion products were then purified, and ligated to the linearized lenti-mU6 vector using T4 DNA ligase (NEB). The same strategy was used to construct lentiviral sgRNAs-PP7 with different target sequences (MajSat and *Akap6*), by using lenti-mU6-sgMajSat-PP7-1 as PCR template and ligation backbone

The sgRNA-PP7-2 targeting Major Satellites was first built into a lentiviral backbone from pSLQ1661-sgMUC4-E3(F+E) (Addgene #51025) using synthesized ultramer oligos. Non-viral sgMajSat-PP7-2 was generated from lentiviral sgMajSat-PP7-2 by PCR amplification, *Bbs*I-*Eco*RI double digestion and ligation to the sgRNA-MS2 backbone that was predigested by *Bbs*I and *Eco*RI.

### sgRNA scaffold sequences and target sequences

The sequence used for the sgRNA-MS2 scaffold is:

5′-N_17−21_GUUUUAGAGCUAGGCCAACAUGAGGAUCACCCAUGUCUGCAGGGCCUAGC AAGUUAAAAUAAGGCUAGUCCGUUAUCAACUUGGCCAACAUGAGGAUCACCCAUGUCUGCAGGGCCAAGUGGCACCGAGUCGGUGCUUUUUUU-3′.

The sequence used for the sgRNA-PP7-1 scaffold is:

5′-N_17−20_GUUUUAGAGCUAUAAGGAGUUUAUAUGGAAACCCUUAUAGCAAGUUAAAAUAAGGC UAGUCCGUUAUCAACUUGGCCUAAGGAGUUUAUAUGGAAACCCUUAGGCCAAGUGGCACCGAGUCGGUGCUUUUUUU-3′.

The sequence used for the sgRNA-PP7-2 scaffold is:

5′-N_17−20_GUUUUAGAGCUAGGAGCAGACGAUAUGGCGUCGCUCCUAGCAAGUUAAAAUA AGGCUAGUCCGUUAUCAACUUGGCCGGAGCAGACGAUAUGGCGUCGCUCCGGCCAAGUGGCACCGAGUCGGTGCUUUUUUU-3′.

The sequence sgRNAs specific for target used in this study can be found in Supplementary Table 1. The sgRNAs targeting *Igh* switch regions were selected by searching for NGGs in the constant region of *Igh* and were chosen based on their level of repetitiveness. The 6-kb repetitive region on the *Akap6* gene was identified using Tandem Repeat Finder (tandem.bu.edu/trf/trf.html)^26^.

### Cell culture

The HEK293T (American Type Culture Collection (ATCC), #3216), Phoenix-ECO (ATCC #CRL-3214) and mouse 3T3 (ATCC #CRL-1658) fibroblasts cell lines were cultured in DMEM with 10% (v/v) FBS, 100 U ml^−1^ penicillin, 100 μg ml^−1^ streptomycin, 1 × GlutaMAX supplement (Gibco), 1 mM sodium pyruvate (Gibco) and 50 μm β-mercaptoethanol. These cells were maintained at 37 °C and 5% CO_2_ in a humidified incubator. ES cells were cultured at 37 °C and 8% CO_2_ in a humidified incubator. The medium for ES cells used was a 1:1 combination of Advanced DMEM/F-12 and Neurobasal medium, with 1 × N2 Supplement, 1 × B27 Supplement, 1 × L-Glutamine, 100 μm β-mercaptoethanol, 10^3^ U ml^−1^ LIF, 3 μM CHIR99021 and 1 μM MEK inhibitor PD0325901. The ES cells used here were derived from an Sv129 ES cell line and have been previously described^27^.

### Delivery of dCas9 coat proteins and sgRNAs

For dCas9 retrovirus production, 20 μg of MSCVLTR-dCas9-Puro plasmid were transfected into 90% confluent Pheonix-ECO cells in 6 cm dishes using calcium phosphate transfection. For coat protein lentivirus production, 3 μg of pMD2.G plasmid, 9 μg of psPAX2 and 12 μg of the lentiviral vector (MCP-EGFP or PCP-mCherry) were co-transfected into HEK 293T cells. At 60 h post-transfection, dCas9 retrovirus and coat protein lentiviruses were harvested and filtered using a 0.45-μm filter (VWR). A virus cocktail containing the different viruses was added to 3T3 cells for 90 min spin infection at 1,150*g*, 30 °C. Infected 3T3 cells were selected with 1 μg ml^−1^ puromycin for 5 days. EGFP-low and mCherry-low puromycin-resistant cells were selected using flow cytometry cell sorting. Stable clonal cell lines were generated by single-cell selection. These were tested using sgMinSat-MS2 and sgMajSat-PP7-1. The clones with high signal-to-noise ratio were selected. One clone (#3-l) was used in all figures.

The dCas9-EGFP 3T3 stable cell line was generated by transducing 3T3 cells with the retroviral plasmid MSCVLTR-dCas9-EGFP-Puro, puromycin selection and cell sorting for cells with low EGFP.

To introduce sgRNAs into the stable clonal cell line, we transiently transfected a total of 5 μg sgRNA(s) (5 μg for a single sgRNA; or 2.5 μg for each of the sgRNA-MS2 and sgRNA-PP7) to 25 × 10^3^ seeded cells with FuGENE (Promega) following the manufacture's instructions. To stably introduce sgRNAs, we used lentiviral transduction as described above for coat proteins.

For dual-colour labelling in mouse ES cells, we infected cells with PCP-mCherry lentivirus and sorted them for mCherry-low population. PCP-mCherry^low^ ES cells were nucleofected with a plasmid cocktail of pCAGGS-dCas9-HA, EF1α-MCP-EGFP and non-viral sgMinSat-MS2 and/or non-viral sgMajSat-PP7. Nucleofection was performed on a Nucleofector 2b device (Program A-023), with Mouse ES Cell Nucleofector Solution (Lonza), based on the manufacturer's instructions.

### FISH validation

After introduction of sgRNAs, cells were seeded on 8-Well Lab Tek II chambered coverglass overnight before imaging and FISH. Before imaging for the MCP-EGFP and PP7-mCherry signals, cells were fixed in the wells in 4% paraformaldehyde for 10 min followed by several washes with 1 × PBS. Image acquisition of fluorescent proteins was done right after fixation and the positions of recorded cells were saved. Cells were then permeabilized with 0.4% Triton (v/v) followed by several washes with PBS. Next cells were denatured for 30 min with 2 M HCl:H_2_O, and washed in ice-cold PBS. 60 μl of hybridization buffer (50 × Denhardt's Solution, 25% Dextran Sulfate in 12.5 × SSPE, 50% formamide) containing γ-Sat-Cy5 and MinSat-Alexa564 probes was applied to cells in each well for 37 °C hybridization overnight. Cells were then washed as follow: 2 × saline sodium citrate (SSC) at 37 °C for 30 min, 2 × SSC at room temperature (RT) for 30 min and 1 × SSC at RT for 30 min. 60 μl prolong gold (Life Technologies) containing DAPI was applied to each well. Image acquisition of FISH signals was performed straight after completion of the FISH. To overlap FISH signals with fluorescent protein signals in the same cells, we imaged the cells that were captured for fluorescent protein signals by referring to the saved positions.

Labelling of probes was performed by nick translation using DNA Pol I (NEB), DNaseI (Life Technologies), dNTPs and fluorophore-conjugated dUTPs. The γ-Sat-Cy5 probe was prepared from a plasmid containing eight copies of the γ-satellite repeat sequence^28^ and directly labelled with dUTP-Cy5. The MinSat-Alexa564 probe was generated by PCR amplifying 3T3 genomic DNA using a pair of primers (5′-CATGGAAAATGATAAAAACC-3 and 5′-CATCTAATATGTTCTACAGTGTGG-3)^13^ and labelling the purified PCR product with dUTP-Alexa564.

Metaphase FISH analyses were performed essentially as before^29^. In detail, colcemid was added for 2 h at 0.1 μg ml^−1^. Cells were then treated with 0.075 M of KCl for 20 min at 37 °C and then washed twice in a cold 3:1 mix of methanol and acetic acid before being dropped onto cold glass slides. Following air-drying, slides were dehydrated in ethanol and denatured in a solution of 70% Formamide in 2 × SSC at 80 °C for 2 min. After a round of dehydration in cold ethanol, slides were hybridized overnight with fluorescently labelled probes. Slides were washed at RT with two 5 min washes of 50% formamide in 0.5 × SSC and then two 5 min washes with 0.5 × SSC. Slides were mounted in Prolong Gold with DAPI (Life Technologies). The probe identifying the 5′ end of *Igh* was generated by labelling of BAC RP24-386J17 with dUTP-Alexa488 and the 3′ of *Igh* was labelled using BAC CT7-199M11 with dUTP-Alexa594. Chromosome 12 was identified using a mouse chromosome 12 paint labelled in Cy3 from Metasystems.

### Image acquisition and analysis

All pictures except where mentioned were acquired with an Applied Precision PersonalDV live-cell imaging system equipped with an Olympus IX-71 inverted microscope, an Olympus Plan-Apo × 60/1.42 oil objective, a CoolSnap HQ2 CCD camera and Nanomotion III Precision Control stage. Images of fluorescent proteins and FISH signals were acquired with the standard DeltaVision filter set (DAPI, FITC, TRITC and Cy5). Cells were captured in *z*-stacks with 0.5 μm separation in Figs 2, 3 and Supplementary Figs 1,2 and 0.4 μm for Fig. 4b and Supplementary Fig. 3. 3D deconvolution was performed using SoftWorx suite under linux OS.

For Fig. 2b and Supplementary Fig. 2, images were acquired using the same microscope settings and exposure time. Based on raw images, the proportion of cells that have Major Satellite signal and also nucleolar signals were calculated.

To analyse images, 3D deconvolution was performed on all images using SoftWorx suite under linux OS. Further analysis was performed with ImageJ software. All figures were generated from the maximum projection of whole-nucleus *z*-stacks. Figures 2a, 3 and 4b and Supplementary Fig. 3 underwent moderate adjustment of balance and contrast to reduce background noise, whereas Fig. 2b and Supplementary Fig. 2 are maximum projections of raw images across nucleus *z*-stacks. For comparison of signal-to-noise ratio, a line was drawn across multiple spots in different sizes, and a ‘Plot Profile' function in ImageJ was used to generate an intensity profile. To calculate the signal-to-background ratio, the highest intensity value of each peak is divided by the averaged intensity of background in between signal peaks.

For long-term live imaging, cells were plated on 8-Well Lab Tek II chambered coverglass, and the culture medium supplemented with 20 mM HEPES before image acquisition. The environment control unit was used to maintain cell growth conditions: 37 °C, 5% CO_2_ and humidity. One *z*-stack (0.8 μm step size, 15 steps) was acquired every 12 min for 10 h. Multiple z stacks were recorded in one experiment. The final images in the video were maximum *z* projection of each time point.

Metaphase spreads (Fig. 4c) were analysed using a Metafer microscope and ISIS software (Metasystems).

### Data availability

All relevant data are available from the authors upon request.

## Additional information

**How to cite this article:** Fu, Y. *et al*. CRISPR-dCas9 and sgRNA scaffolds enable dual-colour live imaging of satellite sequences and repeat-enriched individual loci. *Nat. Commun.* 7:11707 doi: 10.1038/ncomms11707 (2016).

## Supplementary Material

Supplementary Information Supplementary Figures 1-3 and Supplementary Table 1

Supplementary Movie 1 3D projection of a cell labelled at major and minor Satellites

Supplementary Movie 2 3T3 fibroblasts expressing dCas9, MCP-EGFP and PCP-mCherry and transduced with sgMajSat-PP7 and sgMinSat-MS2 were imaged every 12 minutes for 10 hours. Arrow shows which daughter cell is followed after division.
