# Supplementary material for: CRISPR-dCas9 and sgRNA scaffolds enable dual-colour live imaging of satellite sequences and repeat-enriched individual loci
Source: Nat Commun. 2016 May 25;7:11707. doi: 10.1038/ncomms11707 (PMC4894952; doi:10.1038/ncomms11707)
Supplement: Supplementary Information — Supplementary Figures 1-3 and Supplementary Table 1 [file ncomms11707-s1.pdf]

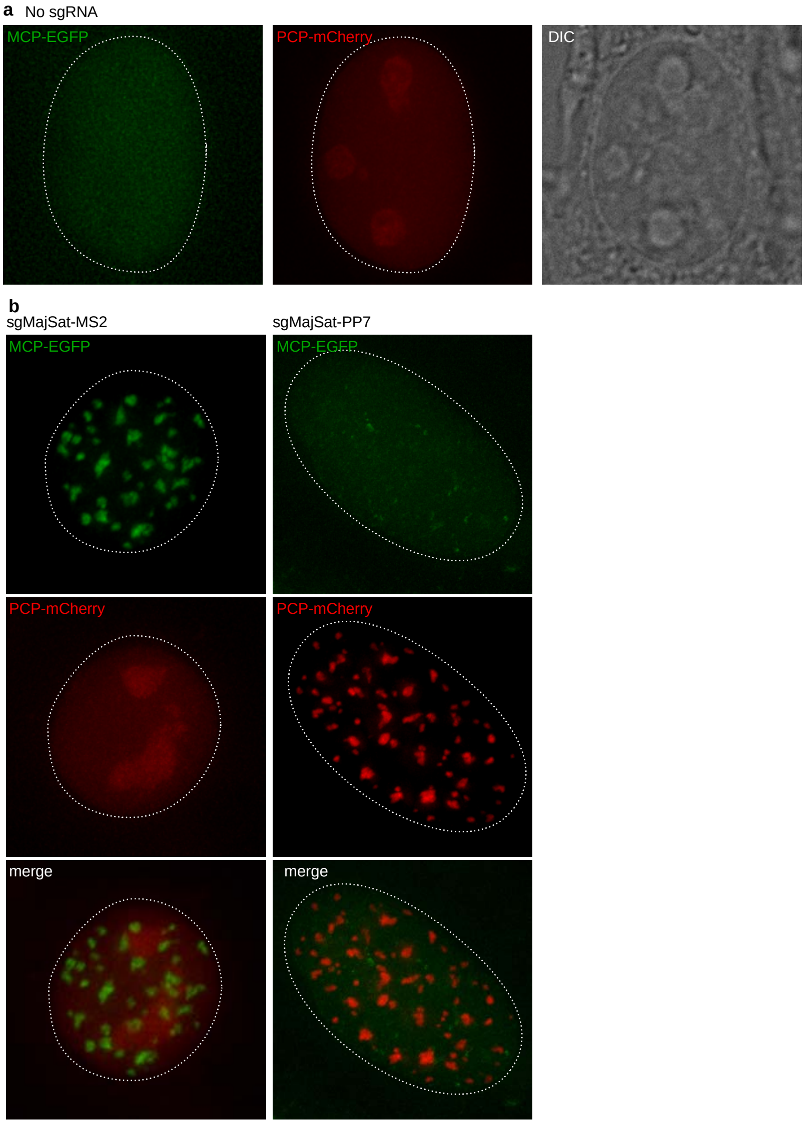

Supplementary Figure 1 – Specificity of fusion fluorescent proteins recruitment to chimeric RNAs

(**a**) Background nuclear expression of MCP-EGFP and PCP-mCherry in 3T3 fibroblasts expressing dCas9 in the absence of sgRNA expression. (**b**) 3T3 fibroblasts expressing dCas9, PCP-mCherry and MCP-EGFP were transfected with sgRNAs targeting major satellites. Transfection of sgRNAs with MS2 stem loops (left) resulted in recruitment of MCP-EGFP to PCH while PCP-mCherry remained localized at the nucleoli. Transfection of sgRNAs with PP7 stem loops (right) resulted in recruitment of

PCP-mCherry (but not MCP-EGFP) to PCH. In the absence of MS2 stem loop expression, MCP-EGFP presents a diffuse nuclear signal.

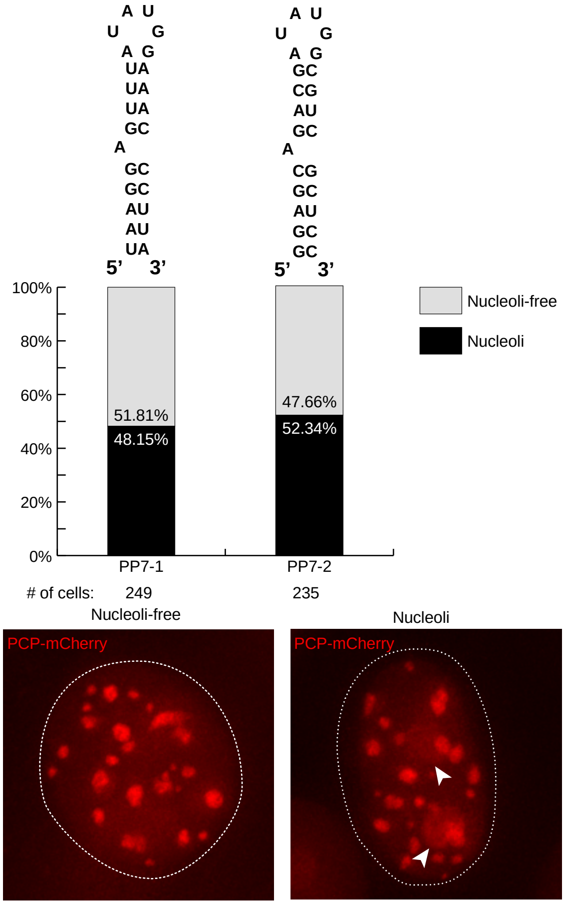

Supplementary Figure 2 – The two versions of chimeric sgRNAs containing PP7 motifs result in equivalent labeling efficiency. Two versions of the PP7 stem loop predicted to form similar secondary structures (composed of different nucleotide sequences) were analyzed (top). We tested which of the two constructs resulted in better recruitment of PCP-mCherry out of the nucleolus into the PCH region. For this we compared the proportion of cells that showed nucleoli-free staining.

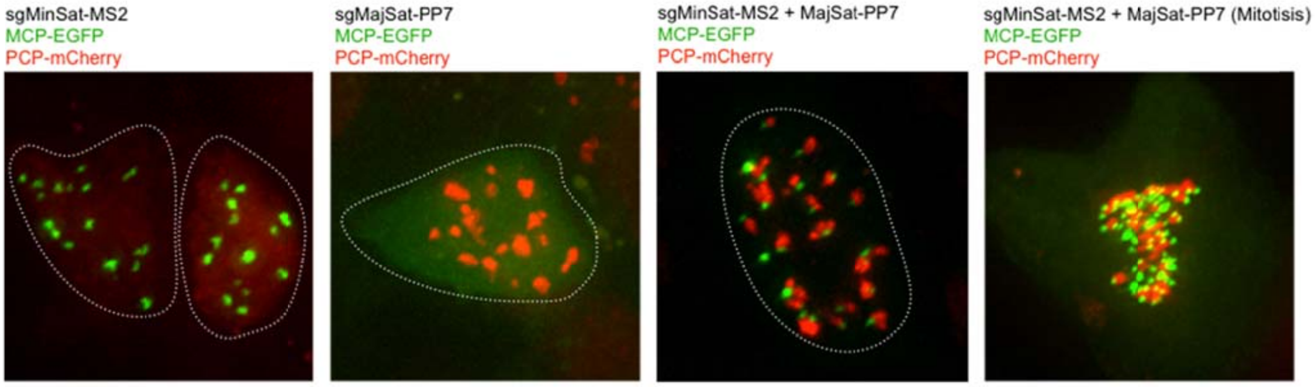

Supplementary Figure 3 - Imaging of minor and major satellite regions in mouse embryonic stem cells. Mouse ES cells were infected with a construct carrying PCP-mCherry and selected for low basal fluorescent protein expression. These cells were then nucleofected with dCas9, MCP-EGFP, sgMajSat-PP7 and sgMinSat-MS2.

Supplementary Table 1 – Sequence of sgRNAs used in this study.

**Region**	**sgRNA sequence**
Major Satellite	CAAGAAAACUGAAAAUCA
Minor Satellite	ACACUGAAAAACACAUUCGU
Akap6	CACAGUGCUCAGGGGACCUGG
Igh Su	UGAGCUGAGCUGAGCUG
Igh Sg1 #1	CACACUUCCACCUGUCC
Igh Sg1 #2	CACACUCCCACCUGUCC
Igh Sg1 #3	CCUGUAGCUGCUCUGCC
Igh Sg2b #1	AGGAGGGAGCUGGGGCAGGU
Igh Sg2b #2	GAUAGGUGGGAGUAUUA
Igh Sg2b #3	AGUCACCCACAGCUGCU
Igh Sg2b #4	UGGGGAUGGUAGGAAUAUGA
Igh Sg2b #5	CCCAUAGCUGCUAGAAC
Igh Sg2b #6	GCUGCCCCAUAGCUGCU
Igh Sg2b #7	AGAUGGUAGGAAUGUGG
Igh Sg2c #1	CCAGGCAGUACAGCUGU
Igh Sg2c #2	AGCUGUGGCUAGGGCCC
